# Binding to DPF-motif by the POB1 EH domain is responsible for POB1-Eps15 interaction

**DOI:** 10.1186/1471-2091-8-29

**Published:** 2007-12-21

**Authors:** Elena Santonico, Simona Panni, Mattia Falconi, Luisa Castagnoli, Gianni Cesareni

**Affiliations:** 1 Department of Biology, University of Rome Tor Vergata, Rome, Italy; 2 Structural Bioinformatics and Computational Biochemistry, Department of Biology, University of Rome Tor Vergata, Rome, Italy

## Abstract

**Background:**

Eps15 homology (EH) domains are protein interaction modules binding to peptides containing Asn-Pro-Phe (NPF) motifs and mediating critical events during endocytosis and signal transduction. The EH domain of POB1 associates with Eps15, a protein characterized by a striking string of DPF triplets, 15 in human and 13 in mouse Eps15, at the C-terminus and lacking the typical EH-binding NPF motif.

**Results:**

By screening a multivalent nonapeptide phage display library we have demonstrated that the EH domain of POB1 has a different recognition specificity since it binds to both NPF and DPF motifs. The region of mouse Eps15 responsible for the interaction with the EH domain of POB1 maps within a 18 amino acid peptide (residues 623–640) that includes three DPF repeats. Finally, mutational analysis in the EH domain of POB1, revealed that several solvent exposed residues, while distal to the binding pocket, mediate specific recognition of binding partners through both hydrophobic and electrostatic contacts.

**Conclusion:**

In the present study we have analysed the binding specificity of the POB1 EH domain. We show that it differs from other EH domains since it interacts with both NPF- and DPF-containing sequences. These unusual binding properties could be attributed to a different conformation of the binding pocket that allows to accommodate negative charges; moreover, we identified a cluster of solvent exposed Lys residues, which are only found in the EH domain of POB1, and influence binding to both NPF and DPF motifs. The characterization of structures of the DPF ligands described in this study and the POB1 EH domain will clearly determine the involvement of the positive patch and the rationalization of our findings.

## Background

The Eps homology (EH) domain is an evolutionary conserved protein interaction module originally identified as a tandem repeat of approximately 100 amino acids at the N-terminus of the proteins Eps15 and Eps15R [1]. When functional information is available, EH-containing proteins are often implicated in regulation of protein transport and membrane traffic [1,2], in actin cytoskeleton organization [2] and in tyrosine kinase signaling pathways [3-6].

Screening with a multivalent nonapeptide phage display library has identified peptides containing an NPF (Asn-Pro-Phe) motif as the preferred ligands of EH domains [3]. While the tripeptide motif is essential for binding, flanking amino acids contribute to modulate binding affinity [7]. A more detailed characterization of several EH domains, by phage display, has revealed that some EH domains may also bind to peptides characterized by different motifs [7]. The NPF-containing peptides are designated class I peptides; class II peptides are characterized by Trp-Trp (WW), Phe-Trp (FW), or Ser-Gly-Trp (SGW) consensus sequences while the EH domain of the yeast protein End3p binds class III peptides containing the His-Ser/Thr-Phe motif [7]. However, till now, no class II or class III motifs have been found to be involved in EH domain recognition in physiological processes.

The structures of five different EH domains have been determined [8-12] and the molecular and structural bases of their binding to class I peptides have been elucidated. The EH domains are formed by two closely associated helix-loop-helix motifs connected by a short antiparallel β-sheet. Comparison of the amino acid sequences of different EH domains indicates that the structurally critical residues involved in the packing of the hydrophobic core are highly conserved throughout the family [11]. Binding to the NPF motif is mediated by a conserved hydrophobic pocket formed by Leu^155^, Leu^165 ^and Trp^169 ^in the second EH domain of Eps15. Mutations in Leu^165 ^and Trp^169 ^were shown to abolish the binding to NPF-containing peptides [7,11].

**Figure 1:**
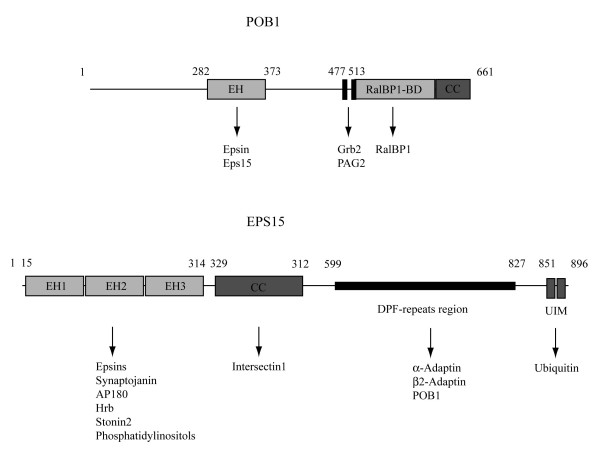
**Domain organization of human POB1 and human Eps15 proteins**. The domain structure of hPOB1 (Swiss Prot entry: Q8NFH8) and of hEps15 (Swiss Prot entry: P42567) and their binding partners are illustrated ([17, 26, 30-36]). Abbreviations: **EH**, Eps15 Homology; **RalBP1-BD**, RalA binding protein 1-binding protein; **CC**, Coiled-Coil region; **UIM**, Ubiquitin Interacting Motif; **Epsin**, Eps-15 Interacting protein; **Eps-15**, Epidermal growth factor receptor pathwat substrate 15; **Grb2**, growth factor receptor-bound protein 2; **PAG2**, Paxillin-associated protein with ARFGAP activity 2; **AP180**, Clathrin coat assembly protein AP180; **Hrb**, HIV-1 Rev-binding protein.

POB1 (Partner of RalBP1) was isolated by yeast two-hybrid screening as a novel interactor of RalBP1 (Ral-binding protein 1), a putative effector protein of Ral [6]. The single EH domain (Fig. 1) is located at the N-terminus of POB1 (residues 282–373 in human POB1) and it is responsible for the interaction with Epsin and Eps15 [13,14], while the C-terminal region has two prolin-rich motifs (residues 477–484 and residues 513–524), which associate with the SH3 domain of Grb2 and with PAG2 [14,15] and a coiled-coil structure that maps inside the region involved in the interaction with RalBP1 [6].

Interestingly, the EH domain of POB1 can bind Eps15, a protein which lacks NPF motifs or other EH-binding motifs (Figure 1). In particular, the POB1 binding-region maps between amino acids 504–745 [14] and it contains 12 DPF (Aspartate-Proline-Phenylalanine) motifs. It has been observed that the EH domain of Reps1, a protein related but not identical to POB1, can bind to a peptide containing a DPF motif [8]; furthermore, Cupers and collaborators reported the presence of tetramers of Eps15 and suggested that they might be stabilized by low-affinity interactions between the N-terminal EH domains and the DPF carboxy-terminal region [16].

The DPF region of Eps15 on the other hand, by binding to the N-terminal "appendage" region of the AP-2 component α-adaptin, is also involved in its recruitment to sites of coated pit assembly [17,18].

In the present work, we have investigated the molecular basis of the unconventional binding specificity of the POB1 EH domain. By screening a multivalent nonapeptide phage display library, we have shown that this domain can bind to both NPF- and DPF-containing peptides. Moreover, we have mapped the region of Eps15 responsible for the interaction with the EH domain of POB1 and compared it with the binding sites for the α-adaptin subunit of the AP-2 complex. In addition, we suggest that also the EH domain region of Eps15 associates with the DPF region, albeit with lower affinity, thus supporting its involvement in the stabilization of Eps15 tetramers.

Finally, based on the NMR structures of both EH domains, we have hypothesized that lysine residues, distal to the binding pocket, are potentially involved in the interaction with Eps15. Altogether, these results show that the peptide recognition specificity of the EH domain of POB1 differs from other EH domains since it interacts with both NPF- and DPF-containing sequences; these unusual binding properties could be attributed to a different conformation of the binding pocket that allows to accommodate negative charges.

## Results

### The EH domain from POB1 binds to NPF- and DPF-containing peptides

**Figure 2:**
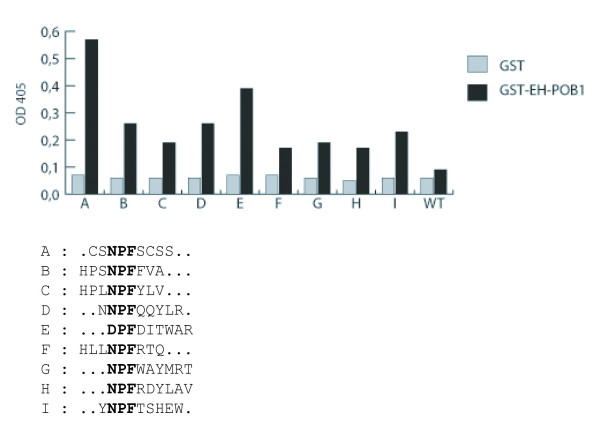
**The EH domain of POB1 binds to both NPF and DPF-containing peptides**. Above: phage clones displaying different peptides were adsorbed to a plastic microtiter plate and incubated with the EH domain of POB1 fused to GST and GST alone as negative control. The bound domain was identified with an anti-GST antibody and a secondary antibody linked to alkaline phosphatase; wt is a phage not exposing any ectopic peptide. Below: sequences of the selected peptides are aligned with respect to the NPF and DPF motifs.

To investigate the binding specificity of the POB1 EH domain, we expressed it as a fusion to glutathione S-transferase (GST) and used it to screen a random nonapeptide phage-display library as previously described [19,20]. 50 clones surviving three panning cycles were further tested by phage ELISA. The 37 phages passing this second test display one of 9 different peptides, 8 of which contain a conventional EH binding motif (NPF: asparagine-proline-phenylalanine) (Fig. 2). The remaining 4 phage clones display the same peptide containing a DPF motif (DPF: aspartate, proline, phenylalanine). The ELISA assay shown in Figure 2 compares the differences in binding affinities between all the selected peptides.

Thus, differently from EH domains whose recognition specificities have been described so far, the POB1 EH domain binds also to a peptide containing a DPF-motif. Furthermore, the selected peptide has a second negatively charged residue at position +1 (aspartate), at variance with the known sequence preferences of EH domains, which dislike negatively charged residues at the positions flanking the NPF tripeptide [7].

Finally, when all the selected NPF peptides are considered together no strong preference can be identified in the positions immediately preceding or following the NPF motif, suggesting that the EH domain of POB1 is less selective when compared to other EH domains described in the literature. This is confirmed by ELISA experiments carried out with purified peptides (not shown).

### POB1 binds DPF motifs in Eps15

**Figure 3:**
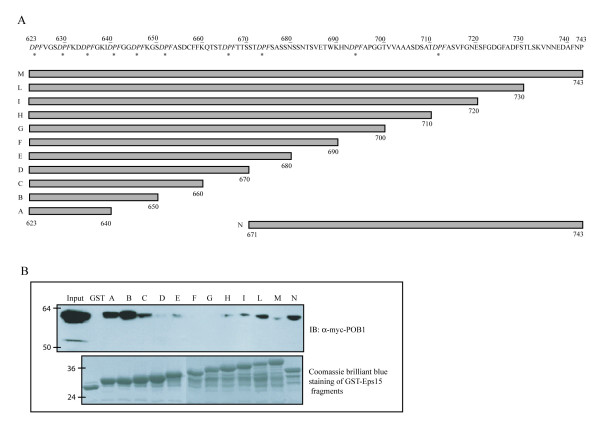
**The EH domain of POB1 binds to an 18 amino acids fragment comprising residues 623–640 and including three DPF**. (A) Schematic representation of the C-terminal DPF-region of mouse Eps15, spanning amino acids 623–750 and comprising 10 DPF tripeptides. The recombinant proteins are progressive COOH-terminal deletions of 10 amino acids fused to the GST. The bars represent the protein fragments that are retained in the recombinant protein. The DPF motifs are indicated by asterisks. (B) Lysates of Hek 293Phoenix expressing Myc-POB1 were incubated with GST or equal amounts of the recombinant proteins bound to glutathione-sepharose beads. Adsorbed proteins were identified with anti-Myc antibody. In the lower panel the GST fusions are visualized with Coomassie staining.

The results so far indicate that the POB1 EH domain can bind DPF peptides displayed on filamentous phage and suggest that this novel binding specificity could be responsible for the recognition of the Eps15 C-terminal domain. To map the Eps15 sequences responsible for binding to POB1 we expressed as GST fusions progressive COOH-terminal (10 amino acids) deletions of the region spanning amino acids 623–743 of mouse Eps15 (Fig. 3A) [18]. Agarose-immobilized GST-Eps15 fusion proteins were tested in a pull-down assay carried out on 293Phoenix cells over-expressing full-length POB1 fused to the Myc epitope. The results reported in Figure 3B indicate that the binding of the POB1 EH domain to the C-terminal region of Eps15 is influenced in a complex manner by the length of the region. Shortening of the 121 amino acid DPF rich fragment initially results in negative modulation of binding (see constructs D, F and G); further shortening, however, restores efficient binding. A C-terminal fragment (N in Fig 3A) is also capable of binding. These results are compatible with the presence of at least two different binding sites with fragment length affecting conformation and, as a consequence, availability of DPF motifs for EH binding.

**Figure 4:**
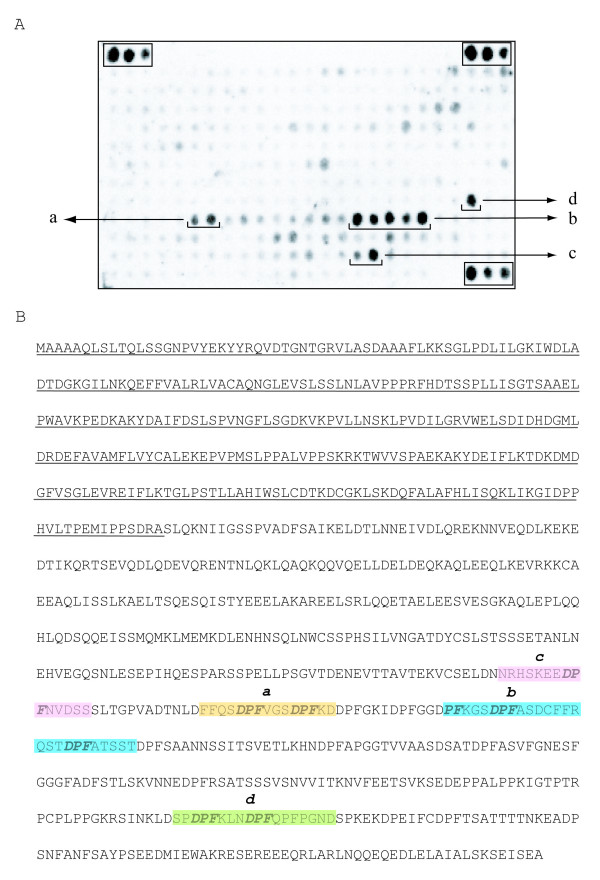
**POB1 associates with DPF-containing peptides in the C-terminus of Eps15**. (A) Full-length human Eps15 was synthesized as 15 amino acid peptides, overlapping by 12 amino acids, on a cellulose membrane using the SPOT synthesis method [21]. The membrane was incubated with the EH domain of POB1 fused to the GST and probed with an anti-GST antibody. The groups of spots considered to be positives are indicated as a, b, c and d. Positives controls, binding to secondary antibodies, are indicated with rectangles. (B) Amino acid sequence of human Eps15. The N-terminal region comprising the three EH domains is underlined, sequences corresponding to the spots considered to be positives are outlined. Residues corresponding to the indicated regions are: a (aa 623–633), b (aa 647–674), c (aa 592–606) and d (aa 796–813).

In order to confirm the EH targets inferred by the pull down experiment, we synthesized the full-length human Eps15 protein as 15 amino acid long peptides, overlapping by 12 amino acids, using the SPOT synthesis method [21]. The membrane was incubated with the EH domain of POB1 fused to GST and probed with an anti-GST antibody (Fig. 4). The results are partially in agreement with the conclusions drawn from the pull-down experiments. POB1_EH binds to DPF peptides in the regions 623–633 (a) and 647–674 (b); these findings are consistent with the pull down results as the sequences (a) and (b) are also present in the C and D constructs (see Fig. 3A). In addition, two more regions, both containing DPF peptides and encompassing respectively amino acids 592–606 (c) and 796–813 (d) also associate to POB1_EH domain. However, both regions, which may contribute to stabilize binding, are outside the fragments used in the pull-down experiment. Finally, the spots encompassing the sequence of the N construct in the GST pull-down fail to associate with the EH domain.

By combining the results from pull down and the PepSpot experiments we conclude that many DPF peptides have the potential of binding the EH domain of POB but most of the Eps15 binding determinants are included in the fragment encompassing amino acids 623–670 and containing five DPF motifs. Furthermore, the two N-terminal DPF peptides included in the region from D_623 _to D_633 _are sufficient to promote binding to POB1_EH. Finally, the results suggest that some of the determinants involved in this interaction might promote a conformation necessary for binding without being involved in the establishment of contacts or, as an alternative, they could act as recognition sites for other binding partners which promote the association.

Cupers and collaborators proposed that Eps15 forms a head to head dimer and that two dimers further associate head to tail to form a tetramer [16]. The tetrameric head to tail structure suggests a possible interaction between the EH domains at the N-terminus of Eps15 and the C-terminal tail which contains 15 DPF repeats.

**Figure 5:**
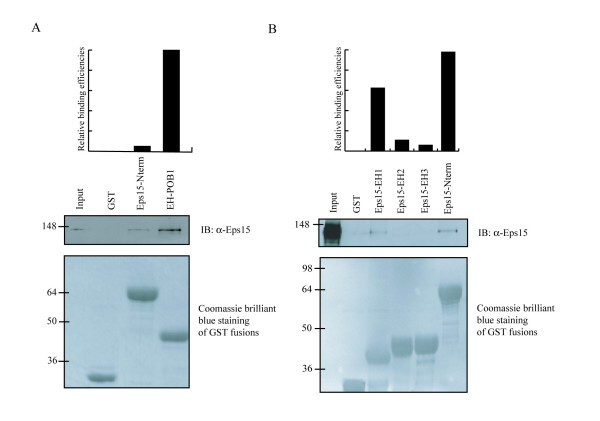
**The amino-terminal EH domain of Eps15 associates with full length Eps15**. (A) The EH domain of POB1 and the N-terminal region of Eps15 comprising three EH domains (EH1-EH2-EH3) were tested in a pull-down experiment from Hek293 to evaluate the relative binding efficiency towards endogenous Eps15. (B) The isolated GST-EH domains (EH1, EH2, EH3) and the N-terminal region of Eps15 were tested in a similar experiment. The input lane corresponds to 0,1% of the lysate. Relative binding efficiencies represent the quantification of the Western blotting using the Image Quant Software. In the lower panel, the GST fusions are visualized by Coomassie staining.

To test this hypothesis, we expressed as GST-fusions the N-terminal region of mouse Eps15 and we compared it with the EH domain of POB1 in a pull-down experiment: 50 μg of purified molecules bound to glutathione-sepharose beads were incubated with 3 mg of cell extract from Hek293. Bound proteins were resolved by SDS-PAGE and analyzed by western-blotting using an anti-Eps15 antibody. The result shown in figure 5A confirms that both the POB1_EH domain and the N-terminal region of Eps15 associates to full length Eps15. A similar experiment was carried out with the isolated GST-EH domains (EH1, EH2, EH3) indicating that the association is mediated by the N-terminal EH domain (EH1) of Eps15 (Fig. 5B).

**Figure 6:**
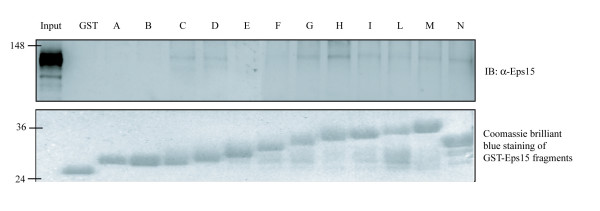
**The DPF region of Eps15 binds to Eps15 in a pull-down experiment**. 50 μg of the Eps15 recombinant proteins spanning amino acids 623–750 and comprising 10 DPF tripeptides, as described in Figure 3, were incubated with a cell extract from Hek293. Bound proteins were resolved by SDS-PAGE and analyzed by western-blotting using an anti-Eps15 antibody.

In order to provide further evidence for the interaction of Eps15 with the N-terminal EH domain, we performed a pull-down assay using recombinant deletion constructs of Eps15, spanning the DPF region. The GST fusions were incubated with a Hek293 cell extract. Affinity purified proteins were resolved by SDS-PAGE and analyzed by western-blotting using an anti-Eps15 antibody to identify endogenous Eps15. The result (Fig. 6) shows an association that involves DPF triplets at the C-terminal end of Eps15. Although these experiments don't definitively prove a direct DPF/EH interaction in Eps15, as we cannot exclude the involvement of accessory proteins, they confirm a plausible role of EH-DPF interaction in the stabilization of Eps15 tetramers, as suggested by other authors [16].

### Lysine residues distal to the binding pocket are involved in POB1 EH/DPF interaction

**Figure 7:**
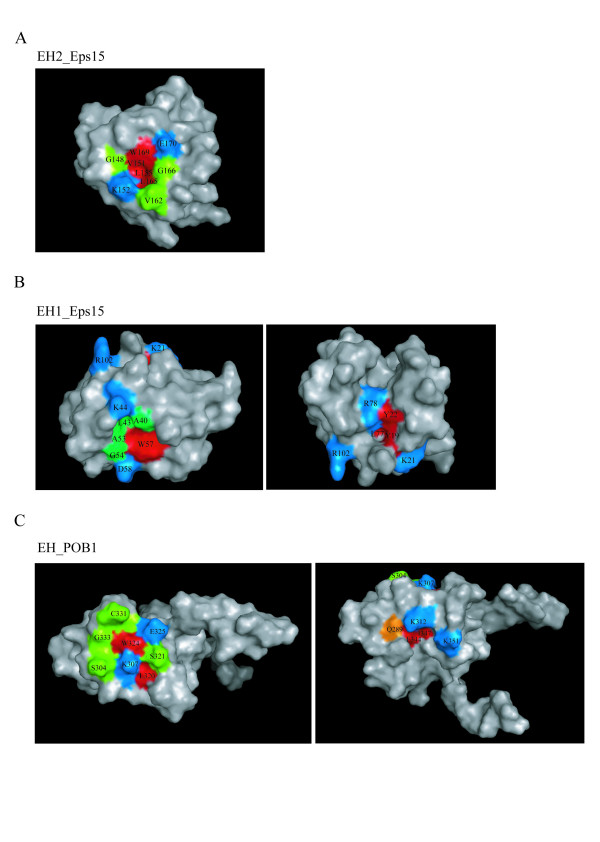
**Structural analysis of the NPF-binding pocket of Eps15 (EH2) and POB1 EH domain**. Molecular surface representations of the NPF-binding pocket of the Eps15 EH2, PDB code: **1EH2 **(A), Eps15 EH1, PDB code: **1QJT **(B) and POB1, PDB code: **1IQ3 **(C) EH domains. Residues in the hydrophobic groove are coloured in red, residues which line the edge of the binding pocket are in green while the gate charged residues are in blue. (B) *Left panel*: representation of the classical binding pocket of Eps15 EH1 domain. *Right panel*: residues distant from the binding pocket, which have been discussed in the text, are mapped on the molecular surface of Eps15. Lys 21 indicates the position of the binding pocket. (C) *Left panel*: representation of the classical binding pocket of POB1 EH domain. *Right panel*: residues mutated in this study are mapped on the molecular surface of POB1. Lys 307 indicates the position of the binding pocket. Lysine residues are coloured in blue, Phe344 and Ile347 in red and Gln289 in orange. Molecular surfaces were generated with PyMol.

NMR studies of the EH2 domain of Eps15, combined with mutational analysis, have identified a patch of hydrophobic residues (Trp169, Val151, Leu155 and Leu165) accommodating the proline in the NPF ligand (Fig. 7A). Residues Gly148, Val162 and Gly166 delineate the edge of the binding pocket where they may contribute to EH recognition specificity. Finally, the side chains of Lys152 and Glu170 are positioned to form a sort of gate flanking the binding groove [9]. It has been suggested that the electrostatic repulsion from the negatively charged side chains in the gate structure may prevent the interaction with a peptide containing an aspartate in place of the asparagine of the NPF motif.

The EH1 domain of Eps15 contains a similar hydrophobic binding pocket as the EH2 domain [12] delimitated by residues Ala40, Leu43, Ala53, Gly54 and Trp57, while Lys44 and Asp58 form the gate of the pocket (Fig. 7B, left panel).

The corresponding residues in the POB1 EH domain are arranged differently [8,22], thus designing a groove with different properties (Fig. 7C, left panel). Some residues such as Phe310 and Ala306 (corresponding to Leu155 and Val151 in EH2_Eps15) are less exposed to the solvent, moreover, the side chain of Lys307 (corresponding to Lys152 in EH2_Eps15) points toward the pocket, partially occluding it. Kim and co-workers determined the solution structure of the EH domain of Reps1 and characterized its binding to different peptides. Based on fast exchange binding analysis, they observed that residues in Reps1 corresponding to Trp324, Glu325 and Ser321 of POB1 do not shift upon addition of a DPF-containing peptide despite these residues showing the largest changes on binding NPF. Furthermore, the addition of DPF peptides affects resonances of residues that are distal to the primary interaction site (Gln240). In POB1_EH the residue corresponding to Gln240 (Gln289) is close to Ile347 and proximal to a positively charged surface patch formed by Lys312 and Lys351 (Fig. 7C, right panel).

**Figure 8:**
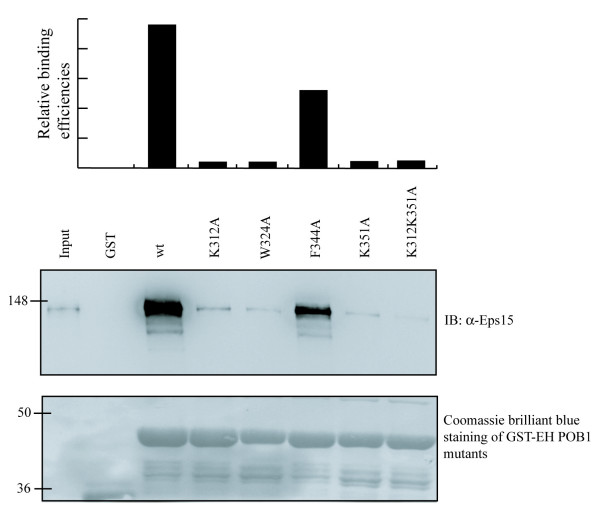
**Mutations in lysine residues distant from the binding pocket affect the interaction of POB1 with Eps15**. Residues K312, W324, F344 and K351 in the POB1-EH domain were changed into Ala and the recombinant proteins were tested in an Eps15 pull-down assay from Hek293. Bound proteins were resolved by SDS-PAGE and analyzed by western-blotting using an anti-Eps15 antibody. In the lower panel, the GST fusions are visualized by Coomassie staining. Relative binding efficiencies represent the quantification of the Western blotting using the Image Quant Software.

These observations could indicate that the topology of the POB1_EH complex bound to a DPF-containing peptide differs somewhat from the topology of a corresponding complex hosting a NPF peptide. Starting from this hypothesis, we investigated the contribution to Eps15 binding of the molecular surface of the POB1_EH domain as defined by the side chains of residues Ile347 and Phe344 and by the positive patch formed by Lys213 and Lys351 in the interaction with Eps15. To this end, we designed four POB1-EH mutant domains containing an Ala side chain at position K312, K351, and F344 respectively. A mutant in the conserved tryptophan (W324A) was also added as a control since this residue was already proven to be involved in NPF recognition. When tested in a pull down assay, mutants K312A, K351A and the double mutant K312A-K351A, similarly to W324A, have lost their ability to bind Eps15 (Fig. 8). By contrast F344A still binds Eps15. A similar result was also obtained when the mutants were tested in an Epsin1 pull down, although POB1_EH binds Epsin1 NPF motifs (data not shown).

Taken together these results suggest that the EH domain of POB1 associates with peptides containing NPF and DPF motifs and that residues that are distal to the previously defined binding pocket may be involved in this recognition.

Finally, the structural analysis (Fig. 7B, right panel) shows that the EH1 domain of Eps15 contains a hydrophobic crevice located in analogous position to the one analyzed by point mutations in the POB1_EH domain. Leu77 and Val80 constitutes the hydrophobic core of the pocket while Tyr19 and Tyr22 are partially solvent exposed and form the edges Finally two positively charged residues, Lys21 and Arg102, surround this groove while Arg78, contiguous to Tyr19, locates the charged guanidinium group toward the centre of the pocket extending the bottom surface. Interestingly, Whitehead and collaborators [12] assert that the mutation of the three tyrosine residues Tyr19, Tyr22, and Tyr23 of mEH1 in Eps15 abolishes its mono-ubiquitination upon stimulation of the cells with EGF, indicating a clear functional role of this region in the regulation of Eps15.

Even if not supported by a mutational analysis, these observations permit to suggest that also for this EH domain residues, distal to the primary interaction site, could be responsible for the EH/DPF interaction and for the "unconventional" recognition specificity.

## Discussion

EH-domain-containing proteins and their interaction partners form a network involved in endocytic transport [23]. They play regulatory roles in endocytic membrane transport events and in actin dynamics. POB1 has been involved in cell migration and receptor endocytosis for its binding the paxillin-associated protein PAG2 [15] and for its interaction with RalBP1, a GTPase-activating proteins (GAPs) for the GTP binding proteins Rac1, CDC42 and Ral [5,6]. Moreover, POB1 was reported to bind directly Epsin and Eps15 with its EH domain [14]. This, in turn, can lead to actin assembly and to the formation of membrane ruffles and actin-rich filopodia [24].

Several studies have pointed out that peptides containing NPF (asparagine-proline-phenylalanine) motifs are consistently found in EH-domain ligands [25]. Structural analyses of the domain ligand complex have demonstrated that the asparagine in the NPF motif is in close contact with a highly conserved tryptophan residue in a hydrophobic pocket in the EH domain surface [9,11]. Mutation of this conserved tryptophan residue dramatically impairs binding. A recent report has implicated a positively charged residue on the opposite side of the NPF binding pocket of some EH domains in phosphatidylinositols binding, thus adding a new function to EH domains [26].

Although EH domains have never been proven to bind to DPF target peptides, the observation that the association between the EH domain of POB1 and Eps15 is mediated by a C-terminal region containing several DPF[14] has lead to the proposal that some EH may be able to bind DPF. In addition it has been suggested that the association between the EH domains of Eps15 and its own DPF rich C-terminus may favour Eps15 multimerization [16].

In the present study we confirm that the POB1 EH domain can bind specific DPF motifs in the C-terminal region of Eps15. This interaction was firstly demonstrated by panning a phage displayed peptide library. With the SPOT synthesis method we have been able to identify many DPF peptides in the C-terminal region of Eps15 with the potential of binding the EH domain of POB, most of them included in the fragment encompassing amino acids 623–670 and containing five DPF motifs. Clearly, a potentially EH binding peptide identified in Eps15 with the SPOT experiment could be buried inside the folded protein and therefore it could be inaccessible to the interaction partner. On the other hand, the previous findings are consistent with the pull-down results as the same sequences also associate to POB1_EH domain. Therefore, the overlap between the results obtained by different approaches confirms that the POB1_EH domain can bind DPF peptides and, more in general, point out the effectiveness of combining the classical pull-down method with the SPOT technology to study protein interactions.

Furthermore, we suggest that a similar association between the first EH domain of Eps15 and its C-terminus could participate in the stabilization of Eps15 tetramers.

The DPF region of Eps15 is also the binding site for the AP-2 complex, the adaptor which connects the clathrin lattice to the plasma membrane and interacts with tyrosine-based signals of several integral membrane proteins [27]. The binding site of AP-2 spans amino acids 668–744 in mouse Eps15, which corresponds to residues 667–739 of the human Eps15 [17]. The EH domain of POB1 binds Eps15 in the region encompassing amino acids 623–670. Thus the POB1 and AP2 binding sites on Eps15 are close but distinct; moreover, neither of these sites coincide with the Crk binding site (residues 768–778 in hEps15) [28], suggesting that the binding of these proteins to Eps15 may not be mutually exclusive. On the other hand the EH of POB1 and the first EH domain of Eps15 both bind to the C-terminal DPF region and compete for the same ligand. It is tempting to speculate that the multimerization of Eps15, stabilized by the interaction between the N-terminal EH and the C-terminal DPF region, may modulate the interaction between POB1 and Eps15.

Structural and mutational analysis of several EH domains clearly demonstrate that the conserved tryptophan at the base of the NPF binding pocket is essential for domain functionality, while residues flanking the binding groove can influence specificity. For example, the EH3 domain of Eps15 binds to a peptide mimicking the FW-internalization motif of MPR; moreover, NMR and mutational analysis unequivocally shows that FW and NPF peptides bind in the same pocket and involve the same residues [10]. Nonetheless, while mutation of residues in the binding site such as Phe252 and Arg249 substantially affect EH3 ligand binding, the corresponding mutants in the residues Leu155 and Lys152 in EH2 do not affect binding [10].

The mutational analysis reported in our manuscript confirms that the conserved tryptophan in the binding pocket of POB1_EH domain is essential for peptide recognition, since the mutation W324A affects the interaction with both NPF- and DPF peptides. In addition we provide evidence that the lysine residues K312 and K351, which are distal to the binding pocket, are involved in this recognition. On the contrary, mutating Phe344 to Ala doesn't affect binding.

## Conclusion

In the present study we have shown that the EH domains of POB1 binds the c-terminus of Eps15 thanks to its affinity for DPF containing peptides. In addition we have identified a cluster of solvent exposed Lys residues, which are only found in the EH domain of POB1, and influence binding to both NPF and DPF motifs. None of the mutants that we have characterized so far show a preferential binding for either NPF or DPF motifs suggesting that the two motifs have a similar binding mode and that both NPF and DPF binding require the hydrophobic pocket characterized in structural studies and the positively charged surface patch characterized in this study. The involvement of the positive patch, either by direct DPF binding or indirectly by modification of the tryptophan binding pocket, cannot be reconciled with the information provided by the structure of the peptide EH complexes described so far and the rationalization of these findings must await the characterization of structures of the DPF ligands described in this study and the POB1 EH domain.

## Methods

### Plasmid vectors and cDNA constructs

The plasmid vectors encoding mouse GST-Eps15 C-terminal constructs were a generous gift from G. Iannolo [18]. The fragments cloned in pGEX expression vector corresponds to the following residues: (A) 623–640, (B) 623–650, (C) 623–660, (D) 623–670, (E) 623–680, (F) 623–690, (G) 623–700, (H) 623–710, (I) 623–720, (L) 623–730, (M) 623–743, (N) 670–743. Cloning of human N-terminal region (EH1-EH2-EH3) has been described previously [7]. The vector encoding human POB1 (Swiss-prot accession number: Q8NFH8) was obtained by PCR amplification of the full-length sequence from a human brain cDNA library; after digestion with BamHI and NotI restriction enzymes the fragment was cloned in pcDNA 3.1 vector containing the Myc epitope tag. The GST fusion protein containing the EH domain of POB1 was obtained by PCR amplification of the region corresponding to residues 266–365, the fragment was digested with BamHI and EcoRI restriction enzymes and cloned in pGEX-2TK.

### Site-specific mutagenesis

The following primers were used to insert the mutations in the EH domain of POB1. CCAAGAACTTCTTCACCGCATCAAAGCTTTCCATTCC for the mutation K312A, AGAACTCTCCTATATAGCGGAGCTTAGTGATGCTG for the mutation W324A, TGAGTTCTGTGCTGCGGCTCATCTCATTGTGGCTC for the mutation F344A, CAATGGGTAGCCGTTCGCCCGAGCCACAATGA for the mutation K351A. Mutations were inserted using the Stratagene's QuikChange Mutagenesis Kit.

### Antibodies

The following primary antibodies were used in the experiments: rabbit anti-Eps15 (Santa Cruz), goat anti-GST (Amersham Bioscience, Inc.), rabbit anti-Myc (Invitrogen) and rabbit anti-Epsin1 (a gift from G. Cestra). Secondary antibodies used in this work were secondary anti-goat monoclonal alkaline phosphatase-conjugated antibody (Sigma), anti-goat IgG peroxidase conjugated (SIGMA), anti-rabbit peroxidase-conjugated IgG and anti-mouse peroxidase-conjugated IgG (Jackson ImmunoResearch).

### Affinity selection

Library construction and panning were performed as described [19,29]. Briefly, 2–20 μg of GST fusion protein bound to glutathione-Sepharose 4B gel (Amersham Pharmacia Biotech) were incubated with 10^10 ^infectious particles from a nonapeptide library. After washing 10times with PBS, 0.5% Tween 20, the bound phages were eluted with 100mM glycine HCl, pH 2.2. After three selection cycles, the binding of isolated clones was confirmed by ELISA.

### Bacteriophage ELISA

Microtiter wells were coated with 10^9 ^particles and incubated with 0.2 μg of GST fusion protein. The wells were then washed 10times with PBS, 0.1Tween 20, and bound protein was detected with anti-GST goat primary antibody (Amersham Pharmacia Biotech) and a secondary anti-goat monoclonal alkaline phosphatase-conjugated antibody (Sigma). Clones with binding activity were selected for further analysis. The sequence of the peptides displayed by positive clones were determined by automatic (ABI PRISM 310Perkin-Elmer) sequencing of phage single-stranded DNA using universal M13-40 primer. For the ELISA with synthetic peptides, biotinylated peptides were bound to microtiter wells coated with 1 μg of streptavidin. Each well was incubated with 0,25 μg of GST-EH domain hybrid protein and the bound domain revealed with an anti GST antibody.

### Cell culture and transfection

293 cells were grown at 37°C in a 5% CO_2 _incubator, in DMEM (Gibco/Invitrogen) supplemented with 10% fetal bovine serum (FBS) (SIGMA-Aldrich) and penicillin and streptomycin (Gibco/Invitrogen). Cells were transiently transfected with Lipofectamine 2000 reagent (Invitrogen) according to manufacture's instructions; alternatively, cells were transiently transfected with the calcium-phosphate method.

### Transient transfection with calcium-phosphate method

One day prior to transfection, 293 cells were plated on 10 cm dish to achieve 60–70% of confluence at the time of the transfection. Briefly, 10 μg of each DNA were diluted in ddH_2_O with 61 μl of 2 M CaCl_2 _to a final volume of 500 μl into a 2 ml tube. Drops of this DNA and CaCl_2 _mixture were added into a 15 ml tube containing 500 μl of HBS 2x, pH 7,05 (50 mM Hepes, 10 mM KCl, 12 mM dextrose, 280 mM NaCl, 1,5 mM Na_2_HPO_4_) while blowing bubbles into the tube with a 2 ml pipet. The mixture was maintained at room temperature for 20 minutes, then the DNA-CaPO_4 _co-precipitated drops were added to the surface of the media containing the cells and the plate was gently swirled to mix. After 8–16 hours the CaPO_4 _containing medium was removed and replaced with normal medium. Transient assays for gene expression in transfected cells were performed 24–72 hours post transfection.

### GST-fusion proteins purification

All constructs were transformed into *E.coli *BL21(DE3) strain. Cultures were grown overnight in LB medium supplemented with ampicillin and used to inoculate fresh medium (v/v 1:100). The culture were subsequently grown to OD_600 _= 0,6 and induced for 4 hours at 30°C with 0,5 mM IPTG. Cells were collected by centrifugation and resuspended in 1/50 of the original volume with STE buffer (10 mM Tris-HCl pH 8, 100 mM NaCl, 1 mM EDTA, Triton 1%) supplemented with protease inhibitor cocktail (SIGMA-Aldrich). For the purification of wild-type and mutated EH domains, cells were resuspended in EH buffer (Tris pH 7,5 50 mM, NaCl 100 mM, CaCl_2 _5 mM, DTT 2 mM, PMSF 1 mM, Triton 1%, Leupeptin 5 μg/ml, Aprotinin 5 μg/ml). Cells were disrupted by sonication and the lysates were clarified by centrifugation. To purify GST-fusion proteins from the extract, the lysates were incubated with glutathione-Sepharose 4B beads (Amersham Bioscience Inc.) for 1 hour at 4°C, then washed 5 times with ice-cold PBS. Were necessary, proteins were eluted from the beads by incubating them with 15 mM glutathione and eluted proteins were subjected to dialysis in cold PBS.

### Pull-down

GST-fusion proteins and GST alone were expressed in bacteria, adsorbed to glutathione-Sepharose beads (Amersham Bioscience, Inc.) and incubated for 2 hours at 4°C with 1 mg of 293 cellular extract in EH buffer. Beads were then washed 3 times with the lysis buffer and bound proteins were electrophoresed on a 10% polyacrylamide gel, transferred onto nitrocellulose membrane and probed with specific antibodies. Detection was performed with the enhanced chemoluminescence Supersignal West Pico Stable Peroxidase Solution (Pierce).

### Peptide array synthesis

The peptide arrays (15 amino acid long) were synthesized on cellulose-(3-amino-2-hydroxy-propyl)-ether (CAPE) membranes, because of a better signal-to-noise ratio in the incubation experiments.

Preparation of CAPE membranes: a 18 × 28 cm Whatman 50 paper (Whatman, Maidstone, United Kingdom) was immersed in a stainless steel dish containing a solution of 400 mg of p-toluenesulfonic acid in methanol (50 ml) and shaken for 3 min. The membrane was removed from the tray and air-dried. Meanwhile a solution of 7.8 g of N-(2,3- epoxypropyl)-phathalimid in dioxane (60 ml) was heated to 80°C in a covered stainless steel dish placed on a shaking platform. Then, a solution of 400 mg of p-toluenesulfonic acid in 5 ml of dioxane was added. The membrane was placed in this solution and shaken at 80°C for 3–5 hours. Afterwards, the membrane was washed three times with 50 ml of dioxane and with ethanol (twice, 50 ml each) and subsequently incubated with a 10% (v/v) solution (50 ml) of hydrazine hydrate (80%) in ethanol for approximately 6 hours. Finally, the membrane was washed twice with ethanol, three times with dimethyl-acetamide, and once again with ethanol (twice, 50 ml each), and dried. The peptide loading of this type of amino-functionalized cellulose membrane is about 120–200 nmol/cm^2^.

Peptides were synthesized according to standard SPOT synthesis protocols (Frank 1992) using an automatic spot synthesizer (Abimed, Langenfeld, Germany) as described in detail (Kramer and Schneider-Mergener 1998).

Incubation with the domain was performed with 10 μg/ml of the GST-fused domain in blocking buffer overnight at 4°C. After washing three times for 10 min with TBS, the anti-GST monoclonal antibody (G1160; Sigma) was added at a concentration of 1 μg/ml in blocking buffer for 2 hours at room temperature. After three washings with TBS (10 minutes each) a POD-labeled anti-mouse mAb (1 μg/ml in blocking buffer) was added and incubated for 1.5 hours at room temperature, followed by washing three times with TBS.

Quantization of peptide-bound domain was carried out using a chemoluminescence substrate and the Lumi-ImagerTM instrument (Roche Diagnostics, Basel, Switzerland). Signal intensities were measured in BLUs (Boheringer Light Units).

## Authors' contributions

ES carried out the affinity selection, the ELISA and pull-down experiments, mutants analysis and drafted the manuscript. SP performed the Pep Spot analysis. MF helped in the structural analysis. LC participated in the design and coordination of the study. GC participated in the design and coordination of the study and drafts the manuscript. All authors read and approved the final manuscript.
